# Supplementary material for: Prevalence and factors associated with experience of corporal punishment in public schools in South Africa
Source: PLoS One. 2021 Aug 12;16(8):e0254503. doi: 10.1371/journal.pone.0254503 (PMC8360577; doi:10.1371/journal.pone.0254503)
Supplement: Attachment — Submitted filename: renamed_da594.docx [file pone.0254503.s001.docx]

**COMMENTS – Manuscript PONE-D-20-20199**

Title: “Prevalence and factors associated with experience of corporal punishment in public schools in South Africa.”

**Important note: This review pertains only to ‘statistical aspects’ of the study and so ‘clinical aspects’ [like medical importance, relevance of the study, ‘clinical significance and implication(s)’ of the whole study, etc.] are to be evaluated [should be assessed] separately/independently. Further please note that any ‘statistical review’ is generally done under the assumption that (such) study specific methodological [as well as execution] issues are perfectly taken care of by the investigator(s). This review is not an exception to that and so does not cover clinical aspects {however, seldom comments are made only if those issues are intimately / scientifically related & intermingle with ‘statistical aspects’ of the study}. Agreed that ‘statistical methods’ are used as just tools here, however, they are vital part of methodology [and so should be given due importance].**

**COMMENTS:** Abstract-Methods section says “3743 grade 8 learners (2118 girls and 1625 boys) from 24 selected public schools in Tshwane, South Africa, enrolled in a cluster randomised controlled trial” my question is if the schools (clusters) are purposively selected, where is randomization? [according to lines 109-111, “All Grade 8 learners in 24 purposively selected English medium State secondary schools within a 50km radius from Pretoria City were eligible to participate in the study”].

Although, it is said in line 178 that [‘All data analysis procedures used took into account the study design’], it is not observed. In this context [authors may already know them, however, not found in the references of this manuscript], here are three good references.

1. Donner Allen and Klar Neil. `Design and Analysis of Cluster Randomization Trial in Health Research’, Oxford University Press Inc., New York, 2000.

2. Bland JM and Kerry SM. ‘Statistics notes: trials randomized in clusters’, Br. Med. Jr., 1997, Volume 315, p600.

3. Kerry SM & Bland JM. ‘Analysis of a trial randomized in clusters’ by in British Medical Journal, Volume 316, 1998, p54.

Also note that

Though the measures/tools used are appropriate [Example - Line 112-3: A survey was conducted using a self-administered questionnaire loaded in personal digital assistants (PDAs)], most of them yield data that are in [at the most] ‘ordinal’ level of measurement [and not in ratio level of measurement for sure {as the score two times higher does not indicate presence of that parameter/phenomenon as double (for example, a Visual Analogue Scales VAS score or say ‘depression’ score)}]. Then application of suitable non-parametric test(s) is/are indicated/advisable [even if distribution may be ‘Gaussian’ (i.e. normal)]. Agreed that there is/are no non-parametric test(s)/technique(s) available to be used as alternative in all situation(s) [suitable / most desired/applicable], but should be used wherever/wherever they are available.

From Table 1 [Description of the factors that influence experience of corporal punishment at school] it appears that many measures used are of this type. I request the authors to also note that (this note is copied and pasted from a standard text book) : Whenever response options ranged from 1=strongly disagree to 4=strongly agree (or ranging from 1 (strongly disagree) to 6 (strongly agree) or from 1=very bad to 3=neither good nor bad to 5=very good), while using a ‘Likert’ scale responses, recoding [like strongly disagree=-2, disagree=-1, neutral=0, agree=1, strongly agree=2] may yield correct and meaningful ‘arithmetic mean’ which is useful not only for comparison but has absolute meaning, in my opinion. Application of any statistical test(s) assume that meaning of entity used (mean, SD, etc) has a particular meaning. Though ‘α’ [alpha] or most other measures of reliability/correlation will remain same, however. Use of non-parametric methods should/may be preferred while dealing with data yielded by any questionnaire/score.

From account given in lines 228-9 [Table 3 shows the relationship between experiencing CP at school and individual measures of the different latent constructs] it is clear that in table-3 {**Bivariate relationship between corporal punishment experience score and a learner’s home environment, attitude and behavior measures.**} first column Coef is ‘Correlation Coefficient’ (mostly Pearson’s)	{LCL is lower & UCL is upper limit of CI I guess, why not given in footnote). Coefficient values are very small, yet most p-values show highly significant results. Is not that contradictory? In this context again it may please be noted that (following note is from a standard text book):

Statistical test usually used to assess significance of Pearson’s ‘Correlation coefficient (r)’ is ‘t’ [where t = { r √ [(n-2) / (1-r^2^)] }for df=n-2, n is sample size] and here Ho is that the population/standard value of ‘r’ is zero. You need r=0.878 to be significant at 5% when n=5 but you need r=0.273 if n=50 & you need only r=0.088 if n=500. ‘P-value’ heavily depends on sample size. Therefore, it is customary to use the (available in most text books on ‘Biostatistics’ or on ‘www/net’) guidelines [very strongly suggesting to consider an absolute value of ‘Correlation coefficient’] for interpreting positive or negative correlations (and do not rely only on corresponding ‘P’-value but also consider an absolute value of ‘Correlation coefficient’). [This argument is equally applicable to non-parametric Spearman’s ‘Correlation coefficient (ρ)’ as well.]

Please look at an absolute value of ‘Correlation coefficient’ in table-3. It is suggested that ‘If r = +.70 or higher Very strong positive relationship’ or ‘-.70 or higher Very strong negative relationship’. In table-3, all ‘Coef’ are below 0.18 [smallest as small as 0.02]. It is not mentioned however that which type of correlation coefficient is used. But in case in the light of the above, interpretation given in lines 228 onwards is questionable.

Moreover, as said in lines 140-145 CP at school is measured by score [It is derived from 2 items: (i) ‘In the past 6 months were you ever beaten by a teacher?’, with a ‘yes or no’ response and (ii) “A teacher might beat you or physically punish you at our school” with responses as strongly disagree, disagree, agree and strongly agree. Our exploratory analysis showed that learners who agreed to statement (ii) were also likely to respond “yes’ to the first statement. We thus created a score from the 2 responses where high score represents higher likelihood of experiencing CP.] and both ‘simple linear regression’ as well as ‘correlation’ {lines 181-3: We used simple linear regression to assess the relationship between experience of CP at school and all individual measures described in Table 1, and also assessed the correlation amongst the measures} assume that (both ‘independent’ & ‘dependent’) variables are continuous.

Surprisingly use of ‘structural equation modelling’ seems to be correct. Account given in lines 184-198 seems perfectly alright. Figures 1,2,3 are alright. Look at the magnate of coefficients in contrast to coefficients reported/displayed in table-3, though they may be different [in nature & purpose].
